# Retinol-binding protein-4 expression marks the short-term mortality of critically ill patients with underlying liver disease: Lipid, but not glucose, matters

**DOI:** 10.1038/s41598-017-03096-y

**Published:** 2017-06-06

**Authors:** Wei-Ting Chen, Mu-Shien Lee, Chia-Lin Chang, Cheng-Tang Chiu, Ming-Ling Chang

**Affiliations:** 1 Liver Research Center, Division of Hepatology, Department of Gastroenterology and Hepatology, Chang Gung Memorial Hospital, Taoyuan, Taiwan; 2 Department of Medicine, College of Medicine, Chang Gung University, Taoyuan, Taiwan

## Abstract

The implications of retinol-binding protein-4 (RBP4) expression in critically ill patients with underlying liver diseases remain unclear. A prospective cohort study involving 200 liver intensive care unit (ICU) patients was conducted, with 274 blood donors as controls. Patient outcomes were assessed using Cox and Kaplan-Meier analyses. Of the 200 ICU patients (mean age: 56.0 yrs), 79.5% were male, 72.5% were cirrhotic, 62% were septic, 29.5% were diabetic, and 29% expired in the ICU (median admission: 7.5 days). ICU patients had lower baseline RBP4 (25.6+/−18.4 vs. 43.8+/−35.0 mg/L, *p* < 0.001) and total cholesterol (TC) levels than controls. The surviving ICU patients had lower baseline international normalized ratios (INRs) of prothrombin time, model for end-stage liver disease (MELD) scores and sepsis rates, but higher estimated glomerular filtration rates (eGFRs) and RBP4 levels than non-surviving patients. eGFRs, INRs and TC levels were independently associated with RBP4 levels. Only surviving patients exhibited significantly increased RBP4 levels after ICU discharge. Baseline RBP4 levels and MELD scores predicted 21-day (≤10 mg/L) and 1-year (≥25) mortality, respectively. In critically ill patients with underlying liver disease, with a link to eGFRs, INRs and TC levels, the baseline RBP4 may serve as a marker for short-term mortality.

## Introduction

Critically ill patients admitted to the intensive care unit (ICU) are at high risk for death. Scoring models or biomarkers predicting the effectiveness of care and clinical outcomes in ICU patients are in demand, but their reliability may be inconsistent. For example, the acute physiology and chronic health evaluation (APACHE) scoring system, a logistic regression model incorporating physiologic and laboratory parameters, is used to benchmark ICU performance, compare disease-specific mortality ratios, and predict individual patient mortality^1^. However, its performance is poor in specific subgroups, such as the surgical abdominal sepsis population^2^. Adipose tissue has emerged as an important endocrine organ through adipokines^3^, which serve as indictors of long-term energy storage and have a profound influence on multi-organ homeostasis^4^. Moreover, most adipokines possess pro- and anti-inflammatory properties and play critical roles in integrating systemic metabolism with immune function. Thus, adipokines are reliable markers for many diseases, including cardiovascular, metabolic and autoimmune diseases, as well as tumor metastasis^5^. The roles of some adipokines have been investigated with respect to the prognoses of critically ill patients^6–8^. Among these adipokines, retinol binding protein 4 (RBP4), a 21-kDa protein that facilitates the transport of hepatic retinol through the circulation to peripheral tissues, caught our attention. Although adipose tissue is an important source of RBP4, the liver is the primary producer of RBP4 with respect to contributing to whole-body retinoid homeostasis^9^, as adipocytes express only approximately 1/5 as much RBP4 messenger RNA as hepatocytes in lean conditions^10^. Retinoids are lipophilic compounds^11^, and approximately 80–90% of all retinoids in the body are stored as retinyl esters (Res) in the hepatic stellate cells (HSCs) of the liver. In addition to Res, HSCs contain droplets of lipids, including triglycerides, cholesteryl esters, cholesterol, phospholipids and free fatty acids. HSC activation is crucial to the development of hepatic fibrosis and presents as lipid droplet release^12^. Elevated retinoic acid-responsive gene expression is associated with elevated hepatic triglyceride levels^13^; thus, lipid metabolism is closely associated with HSC activation and hepatic fibrosis. Serum RBP4 levels are associated with insulin resistance (IR), hypertriglycemia, obesity, metabolic syndrome, diabetes and fatty liver^9, 14–20^. Although serum RBP4 concentrations have been linked to acute mortality in critically ill patients^6^, whether RBP4 is a suitable marker for prognoses and how lipids affect RBP4 expression in patients with underlying liver disease remain unclear. Thus, we conducted a prospective study involving critically ill patients who were admitted to a liver ICU to determine the implications of RBP4 expression in this patient population by comparing this marker with other known prognostic markers.

## Results

### Baseline characteristics

**Figure 1:**
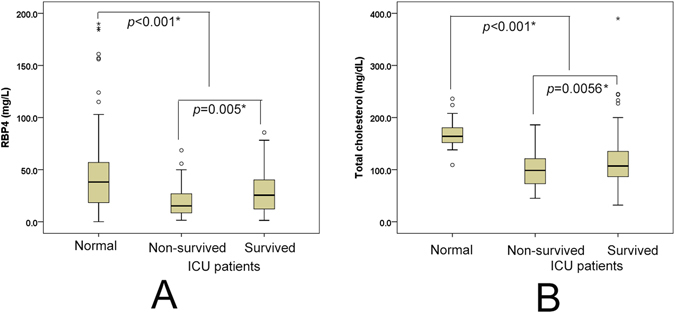
Box-and-whisker plots of the baseline RBP4 (**A**) and TC (**B**) levels in normal controls and liver ICU patients. The outliers are presented as circles or stars. *p < 0.05.

Baseline subject characteristics are presented in Table 1. Among the 200 enrolled liver ICU patients, 159 (79.5%) were male. The mean age was 56.9 yrs. The prevalences of baseline sepsis, cirrhosis, diabetes and hypertension among the ICU patients were 62%, 72.5%, 29.5% and 32%, respectively. The most frequent cause of ICU admission was cirrhosis-related complications (n = 87, 43.5%), followed by acute-on-chronic liver failure (n = 54, 27%), severe acute exacerbations of chronic hepatitis B (n = 31, 15.5%) and ruptured hepatocellular carcinoma (n = 28, 14%). A total of 64 (32%) patients had pulmonary disease, and 105 (52.5%) patients were intubated (for respiratory failure, severe hepatic encephalopathy, or interventional procedure upon consciousness disturbance or unstable hemodynamic status) upon admission. Overall, 58 (29%) of 200 patients expired during the current ICU admission (non-surviving patients). Regarding long-term outcomes, the 30-day, 60-day, 90-day, 180-day and 1-year mortality rates were 35.3%, 45.9%, 47.9%, 52.5% and 56.5%, respectively. ICU patients who survived their illness during their current ICU admissions exhibited lower end-stage liver disease (MELD) scores, shorter ICU stays, lower prevalences of intubation, pulmonary diseases and sepsis, and lower bilirubin (total) levels, international normalized ratios (INRs) for prothrombin time (PT), aspartate aminotransferase (AST) levels, alanine aminotransferase (ALT) levels, aspartate aminotransferase-to-platelet ratio indexes (ARRIs), and neutrophil-to-lymphocyte ratios (NLR)s, but longer hospital stays and higher high-density lipoprotein-cholesterol (HDL-C) levels, estimated glomerular filtration rates (eGFRs) and RBP4 levels (Table 1 and Fig. 1A), than the non-surviving patients. In general, ICU patients had lower baseline RBP4 (Fig. 1A) and total cholesterol (TC) levels (Fig. 1B) than the normal control subjects, irrespective of outcomes.

**Table 1:** Baseline characteristics of the ICU patients [median/mean+/−standard deviation (range)].

Variants	All (n = 200)	Surviving (n = 142)	Non-surviving (n = 58)	*p* values
Sex, (male)*	159 (79.5)	113 (79.6)	46 (79.3)	0.948
Age	56.0/56.9+/−13.8 (32–92)	54.5/56.5+/−13.8 (32–92)	57.5/57.9+/−13.8 (33–88)	0.515
BMI	24.0/24.6+/−4.54 (14.3–39.9)	23.8/24.4+/−4.69(14.3–39.9)	24.4/25.1+/−4.15 (18.6–37.4)	0.299
APACHE IV scores	21.0/20.7+/−9.40 (0–45)	21.0/20.5+/−9.02 (0–40)	20.0/21.2+/−10.3 (0–45)	0.652
MELD scores	25.5/26.4+/−10.10 (7–73)	24.0/23.6+/−8.73 (7–45)	34.0/33.5+/−10.10 (14–73)	<0.001*
ICU days	7.5/10.0+/−8.7 (0–54)	6.0/7.9+/−6.7 (0–32)	14.0/15.2+/−10.8 (0–54)	<0.001*
Hospital days	27.0/36.4+/−33.4 (0–235)	32.0/40.4+/−34.7 (6–235)	21.0/28.1+/−28.4 (0–184)	0.018*
Intubation (yes)*	105 (52.5)	63 (44.4)	42 (72.4)	<0.001*
Diabetes (yes)*	59 (29.5)	41 (28.9)	18 (31)	0.74
Hypertension (yes)*	64 (32)	40 (28.2)	24 (41.4)	0.07
Sepsis (yes)*	124 (62)	78 (54.9)	46 (79.3)	<0.001*
Liver cirrhosis (yes)*	145 (72.5)	102 (71.8)	43 (74.1)	0.689
Pulmonary disease (yes)*	64 (32)	29 (20.4)	35 (60.3)	<0.001*
HOMA-IR	3.32/5.95+/−+/−7.30 (0.12–43.2)	3.39/6.27+/−7.83 (0.2–43.2)	2.68/4.98+/−5.33 (0.12–19.5)	0.388
HbA1C (%)	5.5/5.8+/−1.15 (4.2–11.4)	5.5/5.79+/−1.15 (4.4–11.4)	5.4/5.83+/−1.18 (4.2–9.7)	0.81
C-peptide (ng/dL)	5.6/7.7+/−6.9 (0.4–60.1)	5.9/7.70+/−7.3 (0.4–60.1)	5.1/7.7+/−6.2 (0.4–27.9)	0.968
Uric acid (mg/dL)	5.5/6.3+/−3.9 (0.4–19.8)	5.5/6.5+/−4.0 (0.6–19.8)	5.6/5.9+/−3.6 (0.4–13.6)	0.43
eGFR (mL/min/1.73 m2)	45.0/62.6+/−61.8 (4–118)	33.0/60.5+/−62.6 (9–118)	22.0/35.9+/−38.5 (7–109)	0.008*
TC (mg/dL)	102.0/110.8+/−44.3 (32–390)	107.0/114.9+/−47.7 (32–390)	99.0/100.6+/−32.8 (45–186)	0.056
TG (mg/dL)	75.0/104.3+/−91.1 (18–605)	76.0/108.0+/−100.1 (19–605)	72.5/94.9+/−63.83 (18–279)	0.379
HDL (mg/dL)	15.5/16.8+/−11.0 (2–53)	17.0/18.0+/−11.1 (2–53)	10.0/13.0+/−10.0 (2–40)	0.03*
AST (U/L)	85.5/335.6+/−881.1 (20–6284)	74.0/218.8+/−696.2 (20–6195)	153.0/623.4+/−1183 (27–6284)	0.019*
ALT (U/L)	45.5/206.0+/−512.2 (7–4003)	40.5/129.3+/−288.2 (7–1942)	71.5/394.8+/−814.0 (10–4003)	0.02*
APRI	3.79/14.37+/−37.0 (42–279.5)	3.34/9.40+/−31.5 (0.42–279.5)	7.0/26.6+/−46.3 (0.84–226.4)	0.012*
Albumin (mg %)	2.6/2.6+/−0.457 (0.57–3.7)	2.6/2.7+/−0.48 (0.57–3.62)	2.6/2.5+/−0.50 (0.69–3.7)	0.124
Bilirubin (total) (mg %)	6.3/10.8+/−10.3 (0.2–40.9)	4.0/8.9+/−10.0 (0.2–40.9)	13.5/15.2+/−9.7 (1.5–36.8)	<0.001*
r-GT (IU/L)	73.0/111.7+/−159.5 (7–1600)	74.0/121.0+/−181.7 (11–1600)	69.5/87.0+/−68.4 (7–337)	0.253
HsCRP (mg/L)	29.6/46.3+/−50.7 (0.6–317.3)	32.3/46.8+/−52.1 (0.6–317.3)	25.9/44.7+/−47.2 (1.91–198.1)	0.686
WBC (10^3^/uL)	8.9/11.3+/−10.5 (1.2–116)	8.4/11.2+/−11.4 (2.1–116)	10.0/11.5+/−7.6 (1.2–38.8)	0.842
Platelets (10^3^/uL)	71.0/87.7+/−56.9 (16–317)	76.0/90.8+/−57.5 (16–292)	67.5/80.1+/−55.0 (19–317)	0.209
Hb (g/dL)	8.8/8.9+/−1.8 (5.6–14.8)	8.7/8.9+/−1.7 (5.6–14.8)	8.8/9.0+/−1.8 (5.6–14.5)	0.996
NLR	9.4/15.3+/−19.4 (0.03–97.0)	8.3/13.2+/−17.3 (1.6–95.0)	12.7/20.6+/−23.4 (0.03–97.0)	0.031*
PT (INR)	1.7/1.9+/−0.88 (1.1–7.4)	1.6/1.7+/−0.59 (1.1–4.4)	2.3/2.6+/−1.1 (1.4–7.4)	<0.001*
RBP4 (mg/L)	20.8/25.6+/−18.4 (1.3–85.6)	25.5/27.7+/−19.0 (1.3–85.6)	15.1/20.2+/−15.6 (1.4–68.5)	0.006*

*n (%); BMI: body mass index; MELD: model for end-stage liver disease; APACHE: acute physiology and chronic health evaluation; ICU: intensive care unit; HOMA-IR: homeostatic model assessment for insulin resistance; HbA1c: hemoglobin A1c; TC: total cholesterol; TG: triglyceride; HDL-C: high-density lipoprotein cholesterol; AST: aspartate aminotransferase; ALT: alanine aminotransferase; APRI: aspartate aminotransferase-to-platelet ratio index; r-GT: r-glutamyltransferase; HsCRP: high-sensitivity C-reactive protein; WBC: white blood cell count; NLR: neutrophil-to-lymphocyte ratio; PT: prothrombin time; INR: international normalized ratio; RBP4: retinol-binding protein-4.

### Factors associated with baseline RBP4 levels

**Figure 2:**
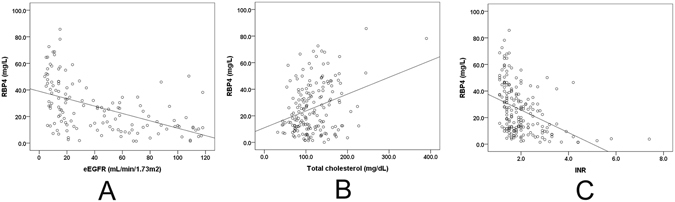
Regression plots for the associations between the baseline RBP4 levels and eGFRs (**A**), TC levels (**B**) and INRs of the prothrombin time (**C**). A, R^2^ = −0.462, *p* < 0.001; B, R^2^ = 0.375, *p* < 0.001; C, R^2^ = −0.404, *p* < 0.001.

The factors associated with baseline RBP4 are listed in Table 2. The univariate analysis demonstrated that age, diabetes, hypertension, C-peptide levels, uric acid levels, eGFRs, TC levels, triglyceride (TG) levels, ALT levels, bilirubin (t) levels, high sensitivity C-reactive protein (Hs-CRP) levels and INRs were associated with baseline RBP4 levels, whereas the multivariate analysis confirmed that the baseline eGFRs, TC levels and INRs were independently associated with the baseline RBP4 levels. The correlations between RBP4 levels and eGFRs (person correlation = −0.458, *p* < 0.001), TC levels (person correlation = 0.371, *p* < 0.001), and INRs (person correlation = −0.404, *p* < 0.001) are presented in Fig. 2.

**Table 2:** Univariate and multivariate analyses of baseline RBP4 levels.

Variants	Univariate analysis: β (95% CI of estimated β)	Univariate analysis: *p* values	Multivariate analysis: β (95% CI of estimated β)	Multivariate analysis: *p* values
Sex (male)	−4.78 (−11.2–1.6)	0.143		
Age (yr)	0.236 (0.051–0.422)	0.013*	0.152 (−0.163–0.466)	0.341
BMI	−0.148 (−0.721–0.425)	0.611		
Diabetes (yes)	7.93 (2.29–13.57)	0.006*	1.78 (−5.3–0.885)	0.618
Hypertension (yes)	8.55 (3.01–14.08)	0.003*	2.88 (−4.1–9.85)	0.412
Sepsis (yes)	3.83 (−1.54–9.2)	0.161		
Liver cirrhosis (yes)	3.02 (−2.75–8.79)	0.303		
Pulmonary disease (yes)	−4.32 (−6.02–5,15)	0.879		
HOMA-IR	−0.21 (−0.69–0.26)	0.374		
HbA1C (%)	0.609 (−1.97–3.18)	0.641		
C-peptide (ng/dL)	0.492 (0.047–0.937)	0.03*	0.219 (0.192–0.619)	0.292
Uric acid (mg/dL)	1.53 (0.807–2.254)	<0.001*	0.669 (−0.368–1.706)	0.203
eGFR (mL/min/1.73 m^2^)	−1.34 (−0.175–0.093)	<0.001*	−0.131 (−0.196–0.065)	<0.001*
TC (mg/dL)	0.16 (0.097–0.223)	<0.001*	0.096 (0.02–0.173)	0.014*
TG (mg/dL)	0.087 (0.058–0.117)	<0.001*	0.023 (−0.016–0.062)	0.244
HDL (mg/dL)	0.091 (−0.224–0.406)	0.57		
AST (U/L)	−0.002 (−0.005–0.001)	0.217		
ALT (U/L)	−0.009 (−0.015–0.002)	0.007*	−0.002 (−0.009–0.005)	0.571
APRI	−0.051 (−0.13–0.027)	0.198		
Albumin (mg %)	−1.56 (−0.825–0.11)	0.645		
Bilirubin (total) (mg %)	−0.27 (−0.53–0.009)	0.043*	0.007 (−0.374–0.387)	0.972
r-GT (IU/L)	0.012 (−0.009–0.033)	0.25		
HsCRP (mg/L)	0.061 (0.009–0.112)	0.022*	0 (−0.052–0.052)	0.991
WBC (10^3^/uL)	0.055 (−0.197–0.306)	0.67		
Platelets (10^3^/uL)	0.035 (−0.011–0.081)	0.14		
Hb (g/dL)	−1.34 (−2.8–0.123)	0.072		
NLR	0.075 (−0.051–0.208)	0.264		
PT (INR)	−8.525 (−11.288–5.762)	<0.001*	−5.05 (−9.2–0.89)	0.018*

RBP4: retinol-binding protein-4; BMI: body mass index; MELD: model for end-stage liver disease; APACHE: acute physiology and chronic health evaluation; ICU: intensive care unit; HOMA-IR: homeostatic model assessment for insulin resistance; TC: total cholesterol; TG: triglyceride; HDL-C: high-density lipoprotein cholesterol; AST: aspartate aminotransferase; ALT: alanine aminotransferase; APRI: aspartate aminotransferase-to-platelet ratio index; r-GT: r-glutamyltransferase; HsCRP: high-sensitivity C-reactive protein; WBC: white blood cell count; NLR: neutrophil-to-lymphocyte ratio; PT: prothrombin time; INR: international normalized ratio.

### Subgroup analyses according to sepsis, cirrhosis and diabetes

Given that septic and cirrhotic patients accounted for the majority of the patients enrolled in this study and that sepsis, pulmonary disease^7^, cirrhosis^5, 6^, and diabetes^21, 22^ may profoundly affect the RBP4 levels, we performed subgroup analyses of the RBP4 levels according to the patients’ sepsis, pulmonary disease, cirrhosis and diabetes statuses. Diabetic (31.87+/−21.33 vs. 23.25+/−16.50 mg/L, *p* = 0.015) patients had higher baseline RBP4 levels than non-diabetic patients. However, there was no difference in the baseline RBP4 levels between the septic and non-septic patients [*p* = 0.161, regardless of adjusting for pulmonary disease (yes: *p* = 0.337; no: *p* = 0.221)], between those with or without pulmonary disease (*p* = 0.879), nor was there a difference in the baseline RBP4 levels between the cirrhotic and non-cirrhotic patients (*p* = 0.303). Regarding the independent predictors of RBP4 levels, higher TC levels (112.1+/−58.2 vs. 106.3+/−37.2, p = 0.039), but lower eGFRs (41.7+/−62.8 vs. 70.1+/−59.7 mL/min, *p* = 0.009), were noted in diabetic patients than in non-diabetic patients. However, no differences in the INR were noted between diabetic and non-diabetic patients.

### Outcome predictors in critically ill patients with underlying liver disease

**Figure 3:**
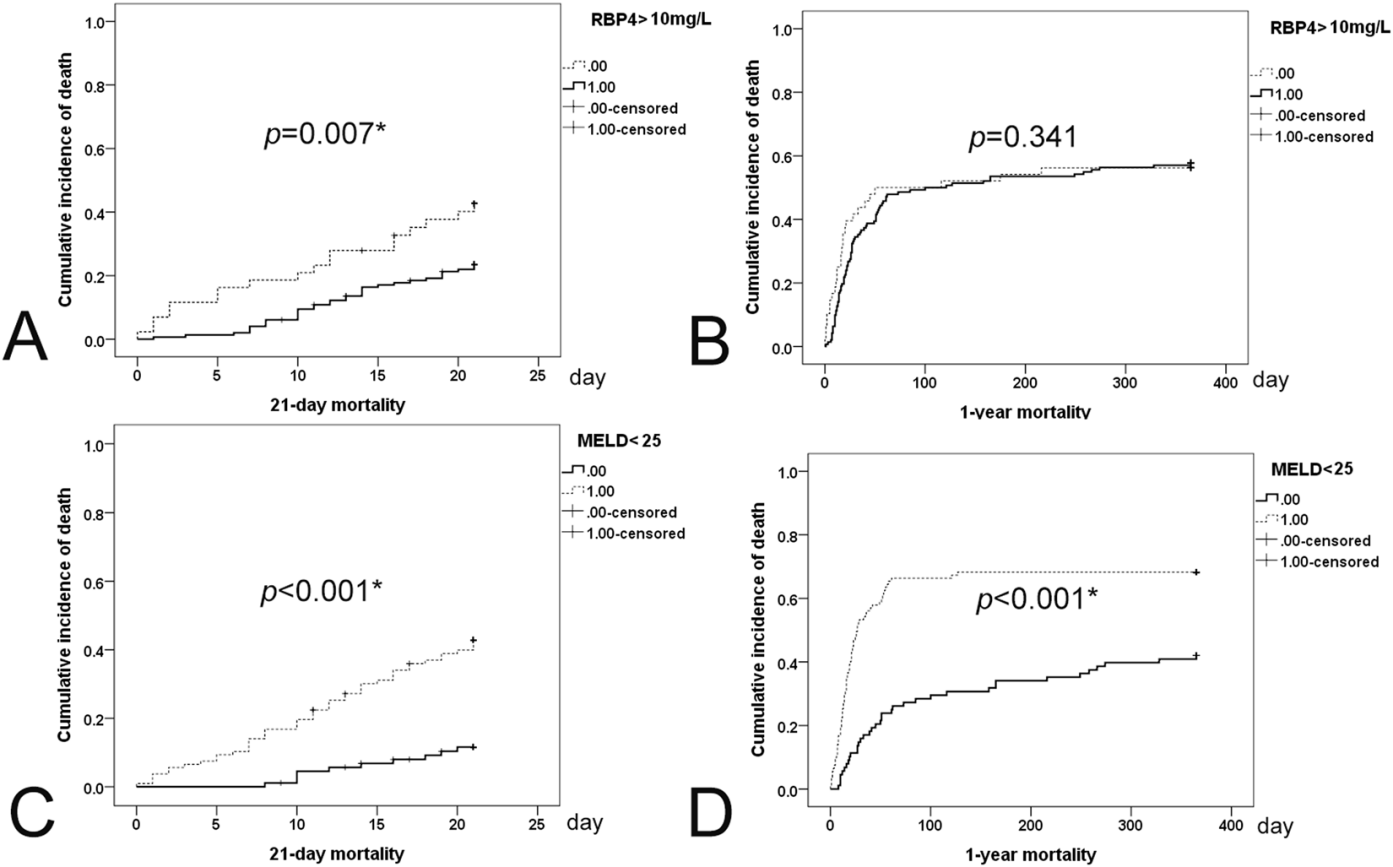
Cumulative incidence of death curves. (**A**) and (**C**), 21-day cumulative incidence of death; (**B**) and (**D**), 1-year cumulative incidence of death. (**A**) and (**B**), dashed lines, baseline RBP4 levels < or =10 mg/L; solid lines, baseline RBP4 levels >10 mg/L. (**C**) and (**D**), dashed lines, baseline MELD scores > or =25; solid lines, baseline MELD scores <25. *p < 0.05.

The usefulness of baseline RBP4 levels and MELD and APACHE IV scores in predicting liver ICU patient outcomes was investigated. The results are presented in Fig. 3. The baseline RBP4 levels could predict short-term (within 21 days of ICU admission) (Fig. 3A), but not long-term (1-year) mortality (Fig. 3B) in critically ill patients with underlying liver diseases. The RBP4 cut-off value for 21-day survival was 10 mg/L. However, no definitive cut-off levels for survival outcomes based on follow-up times longer than 21 days could be determined. Surviving patients (in this ICU admission, 27.3+/−19.1 vs. 33.3+/−21.4 mg/L, *p* = 0.02), but not non-surviving patients (expired during this ICU admission, 20.1+/−17.2 vs. 25.9+/−15.8 mg/L, *p* = 0.078), exhibited significant increases in their RBP4 levels upon discharge from or resuscitation prior to expiring in the ICU. In contrast, baseline MELD scores could predict short-term (Fig. 3C) and long-term (Fig. 3D) mortality up to 1 year, with a cut-off value of 25, whereas APACHE IV scores exhibited a negligible ability to predict both short-term (7-day, *p* = 0.061, 14-day, *p* = 0.186, 21-day, *p* = 0.455, 30-day, *p* = 0.154) and long-term mortality (1-year, *p* = 0.095).

## Discussion

To the best of our knowledge, this is the first prospective study to elucidate the clinical implications of serum RBP4 levels in critically ill patients with underlying liver disease. The following are the most compelling results of this study: (1). Liver ICU patients had lower RBP4 and TC levels than normal controls. Among ICU patients, surviving patients and diabetic patients had higher baseline RBP4 levels than their counterparts (2). eGFRs and INRs were negatively associated with RBP4 levels, whereas TC levels were positively associated with RBP4 levels. (3) Only surviving ICU patients had significantly increased RBP4 levels upon discharge from the ICU. (4) Among liver ICU patients, RBP4 levels could predict short-term mortality (21-day), with a cut-off value of 10 mg/L, whereas baseline MELD scores could predict both short- and long-term (1-year) mortality (cut-off value: 25).

Control subjects had higher baseline RBP4 levels than the liver ICU patients, indicating that stress has a negative impact on RBP4 levels^23^, whereas the surviving liver ICU patients had higher baseline RBP4 levels than the non-surviving patients, suggesting that RBP4 plays a crucial role in restoring vital functions during crises, even in patients with underlying liver disease, which exerts fundamental effects on RBP4 levels^9^. Consistent with this finding, under stress, the body activates an acute phase response to down-regulate negative acute phase proteins including RBP4. Retinoids are RBP4 cargos that regulate cellular proliferation, differentiation, and apoptosis and hence affect immunity maintenance and barrier integrity^24^. The impact of sepsis on the RBP4 levels of critically ill patients may be different in various underlying diseases. In ICU patients with underlying pulmonary disease, lower RBP4 levels were noted in septic patients than in non-septic patients^7^. In contrast, another ICU cohort study demonstrated that sepsis had a negligible impact on RBP4 levels in critically ill patients, regardless of the origin of illness^6^. In our study, no differences in baseline RBP4 levels were noted between the septic and non-septic ICU patients with underlying liver disease, regardless of adjusting for pulmonary disease. Moreover, although cirrhotic critically ill patients had been reported to exhibit lower RBP4 levels than non-cirrhotic critically ill patients^6^, surprisingly, our study did not show such difference. Thus, the impacts of cirrhosis on RBP4 levels may vary between critically ill patients with or without underlying liver diseases. Multivariate analysis demonstrated that both eGFRs and INRs were negatively associated with RBP4 levels, indicating that renal and liver function negatively and positively contribute to serum RBP4 levels, respectively, findings consistent with those of previous studies^9, 25, 26^. Furthermore, although diabetic patients had higher RBP4 levels than non-diabetic patients in the current study, in addition to eGFP and INR levels, TC levels rather than any indicators of glucose metabolism were independently associated with RBP4 levels. Additionally, the above differences in RBP4 levels between diabetic and non-diabetic patients occurred subsequent to increases in TC levels and decreases in the eGFR in diabetic patients rather than being directly driven by diabetes itself. Previous studies have consistently demonstrated that the high RBP4 levels noted in diabetic patients are caused by diminished renal function rather than by altered glucose metabolism^25, 26^. Some studies concluded that glucose metabolism profoundly affects RBP4 levels^6, 21, 22^; however, others have noted a link between lipid metabolism and RBP4 levels^23–29^. Rat studies demonstrated that acute stress had a direct influence on liver lipid metabolism^30^, and human studies demonstrated that low TC levels are associated with a poor prognosis in patients with prolonged sepsis^31^. Moreover, both HDL-C and low-density lipoprotein (LDL-C) levels decreased because of lecithin cholesterol acyltransferase impairment in critical illness^32^. Consistent with these findings, in liver ICU patients, we also showed that HDL-C levels were lower in non-surviving patients than in surviving patients. Collectively, all of the above findings indicate that lipids play a critical role in acute stress. Given that both liver function and RBP4 are closely linked to lipid metabolism^20, 23–29^ and that RBP4 is an acute response protein^6^, we would like to stress that TC levels, but not glucose levels (or glucose metabolism), were independently associated with RBP4 levels in liver ICU patients. Thus, in addition to restoring liver and renal function, supplementing patient diets with adequate TC or related precursors^32^ may up-regulate RBP4-associated pathways and improve the immediate outcomes of critically ill patients with underlying liver disease.

The findings that only the surviving patients exhibited significantly increased RBP4 levels compared with the baseline levels after discharge from the ICU and that baseline RBP4 levels were most effective in predicting short-term mortality confirmed that RBP4 plays a negative acute-phase reactant role in critically ill patients. The negligible roles of the APACHE scores^1^ and the reliabilities of the baseline MELD scores^33^ in predicting both short-term and long-term mortality indicated that only scoring systems focused on liver functional reserve have predictive value with respect to outcomes in critically ill patients with underlying liver diseases.

Because liver and adipose tissue are the major sources of RBP4^9, 10^, the main limitation of this study was its lack of pathological studies of liver and adipose tissue samples. Moreover, the findings documented in the current study should be validated in an independent cohort of liver ICU patients. Future studies of RBP4 incorporating surveys of liver and adipose tissue pathology samples from critically ill patients with underlying liver disease, as well as studies utilizing related cellular or animal models, may be required to elucidate the fundamental molecular mechanisms underlying the findings described herein.

Taken together, critically ill patients with underlying liver disease had lower baseline RBP4 and TC levels than controls. With an association with the eGFR, TC and INR levels, baseline RBP4 levels may serve as a marker for short-term mortality within 21 days of liver ICU admission, with a cut-off level of < or =10 mg/L. In contrast, baseline MELD scores can predict short-term and long-term mortality in liver ICU patients. These findings may facilitate improvements in the outcomes of critically ill patients with underlying liver disease in probing associated metabolic alterations and crucial organ dysfunction.

## Research Design and Methods

### Patients

The study group comprised critically ill subjects aged 18 years or older who were admitted to the liver ICU with underlying diseases, such as chronic hepatitis B, defined as the presence of hepatitis B surface antigens (HBsAg) for >24 weeks^34^; chronic hepatitis C, defined as the presence of documented HCV antibody positivity and detectable HCV RNA for >24 weeks^35^; liver cirrhosis, diagnosed by histologic findings or repeated abdominal ultrasound findings consistent with cirrhosis and supplemented with clinical features such as varices and thrombocytopenia, as described elsewhere^36^; hepatocellular carcinoma; and hepatic failure (complicated by overt hepatic encephalopathy^37^) subsequent to alcohol intoxication and shock. The liver ICU admission criteria included unstable hemodynamic status and single or multiple organ failure in the aforementioned patients. Cirrhosis-related complications were defined as overt hepatic encephalopathy, esophageal variceal bleeding and spontaneous bacterial peritonitis. Overt hepatic encephalopathy was defined as a spectrum of global neurologic deficits in patients with liver dysfunction after the exclusion of brain disease^34, 38^, and was diagnosed and classified as described previously^39^. Esophageal bleeding was determined using a fiberoptic panendoscope. Spontaneous bacterial peritonitis was defined as an ascitic fluid infection without an evident intra-abdominal surgically treatable source^40^ and was diagnosed according to the clinical manifestations and ascitic fluid findings as described previously^41^. Severe acute exacerbation of chronic hepatitis B was defined as an abrupt ALT elevation >5 × the upper limit of the normal range associated with jaundice and a prolonged prothrombin time in patients with chronic hepatitis B^42^. Subjects with human immunodeficiency virus, coronary heart disease, terminal stage of malignancy or solid organ transplant recipients were excluded from the study. Blood donors aged 18 years or older without sepsis, cirrhosis and fatty liver served as the controls.

## Methods

A total of 200 critically ill patients were consecutively recruited from a liver disease ICU at a tertiary referral center between November 2014 and September 2016. We followed the clinical courses of these patients after their discharge from the ICU by contacting them and/or their primary care physicians up to 1 year after they entered this study. Although the definition of sepsis had been revised according to the Sequential (Sepsis-related) Organ Failure Assessment score in 2016^43^, the current study enrolled patients during 2014–2016. Thus for consistency, the patients in this study were classified as septic or non-septic according to the previous guidelines (systemic inflammatory response syndrome criteria)^44^. Pulmonary diseases including pneumonia, chronic obstructive pulmonary disease, acute respiratory distress syndrome and lung cancer were also considered for the statistical analyses. A total of 274 blood donors [205 (74.8%) males; age: 48.68+/−16.85 y; body mass index (BMI): 22.27+/−2.95; ALT: 18.01+/−11.3 IU/mL] were enrolled as controls. We evaluated several baseline factors in all of the enrolled ICU patients, including sex, age, BMI, systolic and diastolic blood pressure, cirrhosis, glucose levels, insulin levels, homeostasis model assessment-estimated insulin resistance (HOMA-IR) [fasting insulin (μU/mL) × fasting glucose (mmol/L)/22.5] levels, glycated hemoglobin (hemoglobin A1c, or HbA1c) levels, C-peptide levels, uric acid levels, creatine levels, eGFRs, AST levels, ALT levels, APRIs, total bilirubin levels, r-glutamyl transpeptidase levels, TC levels, TG levels, HDL-C levels, LDL-C levels, HsCRP levels, white blood cell counts, hemoglobin levels, neutrophil percentages, lymphocyte percentages, NLRs, platelet counts, INRs for prothrombin time and RBP4 levels (R&D Systems, MN, USA), upon their admission to the ICU using fasting serum samples. Illness severity was accessed using the APACHE IV and MELD scores. RBP4 levels were evaluated again in the ICU patients upon discharge from or following expiration in the ICU. Abdominal ultrasound and dynamic computed tomography studies were performed to determine if cirrhosis or hepatocellular carcinoma was present.

### Materials

Biochemical tests were performed in the hospital clinical pathology laboratory using routine automated techniques. Serum hepatitis markers, including HBsAg and HBeAg antibody levels and anti-HDV antibody levels, were assayed using radioimmunoassay kits (Abbott Diagnostics, North Chicago, IL, USA), and anti-HCV antibody levels were assayed using a commercial third-generation enzyme immunoassay (Axsym HCV, version 3 Abbott Diagnostics, North Chicago, IL, USA).

### Statistical Analyses

All statistical analyses were performed using the Statistical Package for the Social Sciences (SPSS package version 21, SPSS Inc., Chicago, USA) software. For the between-group comparisons, continuous variables were analyzed using the Student’s *t*-test or the non-parametric test (Mann-Whitney U), whereas categorical variables were analyzed using the chi-squared test or Fisher’s exact test when appropriate. The continuous variables were expressed as the mean+/− standard deviation (SD) and the median (range), and the categorical variables were expressed as the number (No.) and percentage (%). Correlations between variables were analyzed using Spearman’s correlation tests. Kaplan-Meier and Cox regression analyses were performed to assess the relationships between the various variables and patient outcomes. The co-linearity of the various variables was determined via a linear regression. Variables found to be significant in the linear regression analysis were included in the multivariate Cox regression models, and univariate and multivariate linear or logistic regression models were used to assess the relationships between the various dependent and independent variables. Paired t-tests were used to compare the variables assessed upon ICU admission and discharge within the same individual. Based on the results of the uni- and multivariate Cox regression analyses, Kaplan-Meier curves were generated and log-rank test calculations were performed to evaluate the different RBP4 (round figures, 9–19 mg/L) and MELD score cut-off values (round figures, 14–35). The optimal cut-off values were selected based on the results of the Kaplan-Meier analyses and log-rank tests. Statistical significance was defined at the 5% level based on the results of two-tailed tests of the null hypothesis.

### Institutional Review Board

The study protocol conformed to the ethical guidelines of the 1975 Declaration of Helsinki and was approved by the Chang Gung Memorial Hospital institutional review board. All subjects provided written informed consent to participate in this study.
